# Evaluation of the Efficacy of Serum Lactate-to-Albumin Ratio as a Prognostic Marker for Sepsis and Its Comparison With the Sequential Organ Failure Assessment (SOFA) Score

**DOI:** 10.7759/cureus.105549

**Published:** 2026-03-20

**Authors:** Gowtham Naidu Balle, Siddanagouda M Biradar, Anuja M Kadagud

**Affiliations:** 1 General Medicine, Shri B.M. Patil Medical College, Hospital and Research Centre, BLDE (Deemed to be University), Vijayapura, IND

**Keywords:** lactate-to-albumin ratio, mortality, prognostic biomarker, sepsis, sofa score

## Abstract

Introduction: Sepsis is a condition resulting from an abnormal host response to infection that leads to acute organ dysfunction. Early identification of patients at high risk of adverse outcomes is essential. This study aimed to evaluate the prognostic value of the serum lactate-to-albumin (L/A) ratio for sepsis and compare its performance with the Sequential Organ Failure Assessment (SOFA) score in predicting clinical outcomes.

Materials and methods: This prospective observational study was conducted at a tertiary care centre from June 2024 to December 2025 and included 90 adult patients diagnosed with sepsis. Serum lactate, serum albumin, and the SOFA scores were recorded on day one and day three. Patients were followed until discharge or in-hospital death. The primary endpoint was in-hospital mortality, while secondary endpoints included the requirement for mechanical ventilation, the need for vasopressor therapy, and length of hospital stay. Statistical analysis was performed using SPSS Statistics version 26 (IBM Corp., Armonk, NY, USA). Continuous variables were expressed as mean ± standard deviation and compared using the Mann-Whitney U test or the Wilcoxon signed-rank test, while categorical variables were analysed using the chi-square test. Receiver operating characteristic (ROC) curve analysis was performed to evaluate predictive ability.

Results: Mortality was seen in 42 (46.7%) patients, with a predominance of those aged 61 to 80 years (41, 45.6%) and of the male gender (55, 61.1%). Non-survivors had significantly higher SOFA scores. While the baseline L/A ratio was not significant (p = 0.625), the day three L/A ratio and its change were significantly higher in non-survivors (p < 0.001). On day three, 33 (36.7%) patients required mechanical ventilation and 40 (44.5%) required vasopressors; higher L/A ratios were significantly associated with these outcomes (p ≤ 0.001). The ROC analysis showed strong predictive ability for SOFA day three (area under the curve (AUC) 0.907) and good performance for L/A day three (AUC 0.813).

Conclusion: Serial measurement of the L/A ratio provides meaningful prognostic information comparable to the SOFA score and may serve as a practical risk stratification tool for sepsis.

## Introduction

Sepsis remains a major cause of morbidity and mortality, accounting for an increasing proportion of ICU admissions and deaths [1,2]. The heterogeneity of clinical presentation and the rapid progression to multi-organ failure in severe cases make early identification and accurate risk stratification essential [3]. Sepsis is defined as life-threatening organ dysfunction caused by a dysregulated host response to infection, emphasizing the importance of structured assessment tools for severity evaluation [4].

The SOFA score quantifies organ dysfunction across various systems, and higher SOFA scores correlate with increased mortality and are widely used for prognosis in ICUs [5,6]. However, calculation of the SOFA score requires multiple laboratory parameters and clinical variables, some of which may not be readily available in emergency or resource-limited settings [7]. This has stimulated interest in simpler, rapidly obtainable biomarkers that can reliably predict outcomes while maintaining clinical accuracy.

Serum lactate is a well-established marker of tissue hypoperfusion and cellular metabolic stress in critically ill patients [8]. Elevated lactate levels in sepsis are associated with impaired oxygen utilization, mitochondrial dysfunction, and reduced clearance secondary to hepatic or renal impairment [9]. Conversely, serum albumin, the most abundant plasma protein, reflects nutritional status, hepatic synthetic function, and systemic inflammatory burden [10]. Hypoalbuminemia is common in sepsis and has been independently associated with increased mortality, prolonged hospital stay, and greater need for organ support [11]. While both lactate and albumin individually provide prognostic information, serum lactate alone may be influenced by several factors such as hepatic dysfunction, medications, and altered metabolic states, which may limit its prognostic accuracy in isolation [8].

Recently, composite biomarker ratios have gained attention for integrating distinct pathophysiological pathways into a single prognostic index. The lactate-to-albumin (L/A) ratio combines a marker of tissue hypoxia and metabolic stress with an indicator of inflammatory severity and hepatic function, potentially offering enhanced risk stratification compared to either parameter alone [12]. Previous studies have also suggested that combining lactate with albumin may improve prognostic performance compared with lactate alone, particularly in critically ill patients with sepsis. Hence, the present study aimed to assess the efficacy of the serum L/A ratio by comparing it with the SOFA score and evaluating its association with clinical outcomes.

## Materials and methods

This prospective observational study was conducted at a tertiary care center in Vijayapura, Karnataka, India, from June 2024 to December 2025 and was approved by the Ethical Committee of Shri B.M. Patil Medical College, Hospital, and Research Centre of BLDE (Deemed to be University) (approval no. BLDE(DU)/IEC-SBMPMC/050/2023-24). Patients were recruited from the medical ICU of the tertiary care hospital. The study population comprised adult patients with a diagnosis of sepsis.

Sepsis was identified as a suspected or documented infection accompanied by acute organ dysfunction. Screening parameters included respiratory rate ≥22/min, Glasgow Coma Scale (GCS) <15, or systolic blood pressure ≤100 mmHg, followed by confirmation using organ dysfunction assessment [4]. Patients aged above 18 years who fulfilled the diagnostic criteria were included. Patients with chronic liver disease, chronic kidney disease, or nephrotic syndrome were excluded to minimize confounding effects on serum albumin and lactate levels. The primary outcome of the study was in-hospital mortality, while secondary outcomes included the requirement for mechanical ventilation, vasopressor therapy, and duration of hospital stay.

The sample size was calculated based on the existing literature's -0.52 correlation between platelet count and L/A ratio [13]. Using a two-tailed exact correlation bivariate normal model with an alpha error probability of 0.01 and statistical power of 99%, the minimum sample size was calculated as 75. The calculated exact power was 0.9901023, with a critical correlation coefficient (r) range of −0.2957139 to 0.2957139.

Upon admission, demographic details, including age and gender, were recorded. A detailed clinical history was obtained, and an examination was performed. Baseline vitals and laboratory investigations were noted. Additional investigations such as electrocardiography, two-dimensional echocardiography, and ultrasonography of the abdomen and pelvis were performed when clinically indicated. The SOFA score was calculated for each patient using standard criteria assessing respiratory, coagulation, hepatic, cardiovascular, neurological, and renal parameters [5]. Patients were evaluated on day one and day three of hospitalization, and both L/A ratio and SOFA score were recorded serially to assess dynamic changes. The day-one values represented baseline severity at admission, while day-three measurements were selected to evaluate early changes in clinical status and response to treatment during the initial phase of sepsis management. Differences between day one and day three values (SOFA difference and L/A difference) were calculated to evaluate the biomarker trajectory. No predefined cutoff value for the L/A ratio was used; instead, its prognostic performance was assessed using receiver operating characteristic (ROC) curve analysis.

Data were entered into Microsoft Excel (Microsoft Corp., Redmond, WA, USA) and analyzed using SPSS Statistics version 26 (IBM Corp., Armonk, NY, USA). Continuous variables are shown as mean ± standard deviation and were analyzed using the Wilcoxon Signed Rank test, Mann-Whitney U test, or the Kruskal-Wallis test. Categorical variables are shown as frequencies and percentages and were analyzed using the chi-square test. A p-value <0.05 was considered statistically significant.

## Results

The study included 90 patients with a mean age of 59.48 ± 15.26 years. The majority were aged between 61 and 80 years (41, 45.6%), followed by those aged 41 to 60 years (36, 40.0%), indicating a predominance of middle-aged and elderly individuals. Males constituted 61.1% (55), while females were 38.9% (35), showing a preponderance of males (Table 1).

**Table 1:** Baseline demographic characteristics (total n = 90) Categorical variables such as age groups and gender are expressed as numbers (n) and percentages. Continuous data (age) is summarized as mean ± SD. No inferential statistical test was applied, as this data provides only descriptive baseline characteristics.

Variable	Category	N (%)
Age (years)	17-20	3 (3.3)
	21-40	6 (6.7)
	41-60	36 (40.0)
	61-80	41 (45.6)
	>80	4 (4.4)
	Mean ± SD	59.48 ± 15.26
Gender	Male	55 (61.1)
	Female	35 (38.9)

There was no statistically significant difference in SOFA score (p = 0.800) or L/A ratio (p = 0.354) between day one and day three (Table 2). Between day one and day three, there was a significant reduction in WBC count, serum lactate, serum albumin, and serum creatinine. The partial pressure of oxygen (PaO₂)/fraction of inspired oxygen (FiO₂) ratio and mean arterial pressure improved significantly (p = 0.016 and p = 0.012, respectively) (Table 3).

**Table 2:** SOFA score and L/A ratio distribution on days one and three This table compares the distribution of the SOFA score and L/A ratio between day one and day three. Categorical distributions are presented as numbers (n) and percentages, and continuous variables are summarized as mean ± SD. The Wilcoxon Signed Rank Test was used to compare paired non-parametric data between the two time points, and corresponding Z values and p-values are provided. A p-value < 0.05 was considered statistically significant. SOFA: Sequential Organ Failure Assessment; L/A: Lactate/albumin

Variable	Category	Day one, n (%)	Day three, n (%)	Z value	p-value
SOFA score	0-6	44 (48.9)	50 (55.6)	-0.254	0.800
	7-9	22 (24.4)	10 (11.1)		
	10-14	22 (24.4)	19 (21.1)		
	>14	2 (2.2)	11 (12.2)		
	Mean ± SD	7.48 ± 3.18	7.16 ± 5.20		
L/A ratio	<0.5	5 (5.6)	21 (23.3)	-0.928	0.354
	0.5-1.0	69 (76.7)	50 (55.6)		
	1.01-2.0	16 (17.8)	14 (15.6)		
	>2.0	0 (0.0)	5 (5.6)		
	Mean ± SD	1.12 ± 0.47	1.07 ± 0.92		

**Table 3:** Comparison of clinical parameters on days one and three The Wilcoxon Signed Rank Test was applied. *A p-value < 0.05 was considered statistically significant. PaO₂: Partial pressure of arterial oxygen; FiO₂: Fraction of inspired oxygen ratio; MAP: Mean arterial pressure; GCS: Glasgow Coma Scale

Parameter	Day one (mean ± SD)	Day three (mean ± SD)	Z value	p-value
WBC (cells/µL)	21152 ± 24508	19121 ± 19974	–2.575	0.010*
PaO₂/FiO₂	214.66 ± 119.28	254.20 ± 137.23	–2.402	0.016*
Platelets (cells/µL)	255144 ± 157449	220533 ± 153159	–2.608	0.009*
Bilirubin (mg/dL)	1.47 ± 1.01	1.57 ± 1.54	–0.248	0.804
MAP (mmHg)	80.63 ± 17.10	85.89 ± 13.12	–2.504	0.012*
GCS	12.56 ± 3.10	11.97 ± 4.14	–1.444	0.149
Creatinine (mg/dL)	2.12 ± 1.93	1.80 ± 1.56	–2.468	0.014*
Serum lactate (mmol/L)	2.79 ± 0.97	2.43 ± 1.66	–2.736	0.006*
Serum albumin (g/dL)	2.79 ± 0.69	2.49 ± 0.58	–5.258	<0.001*

Most patients had a hospital stay <14 days 77(85.6%), while 13(14.4%) stayed >14 days. The overall mortality rate was 42 (46.7%), and 48 (53.3%) patients were discharged (Table 4). Non-survivors had significantly higher SOFA scores. The L/A ratio on day one was not significantly different (p = 0.625). However, the L/A ratio on day 3 and the change in L/A ratio were significantly higher in non-survivors (p < 0.001), indicating better prognostic value of serial measurements (Table 5).

**Table 4:** Duration of hospital stay and final outcome Data are presented as frequency (n) and percentage (%). No inferential statistical test was applied, as the data provide descriptive outcome measures.

Variable	Category	N (%)
Hospital stay	<14 days	77 (85.6)
	>14 days	13 (14.4)
Final outcome	Death	42 (46.7)
	Discharge	48 (53.3)

**Table 5:** Comparison of SOFA scores and L/A ratios between survivors and non-survivors The Mann–Whitney U test was applied. *A p-value < 0.05 was considered statistically significant. SOFA: Sequential organ failure assessment; L/A: Lactate/albumin

Variable	Death (total n=42), mean ± SD	Discharge (total n=48), mean ± SD	U value	p-value
L/A ratio 1	1.11 ± 0.53	1.00 ± 0.35	947.5	0.625
L/A ratio 2	1.57 ± 0.99	0.62 ± 0.30	238.5	<0.001*
L/A difference	+0.46 ± 0.75	–0.37 ± 0.29	190.0	<0.001*
SOFA score 1	8.62 ± 3.72	6.48 ± 2.21	640.5	0.003*
SOFA dcore 2	10.95 ± 4.31	3.83 ± 3.29	187.0	<0.001*
SOFA difference	+2.33 ± 3.23	–2.65 ± 2.16	220.5	<0.001*

**Figure 1:**
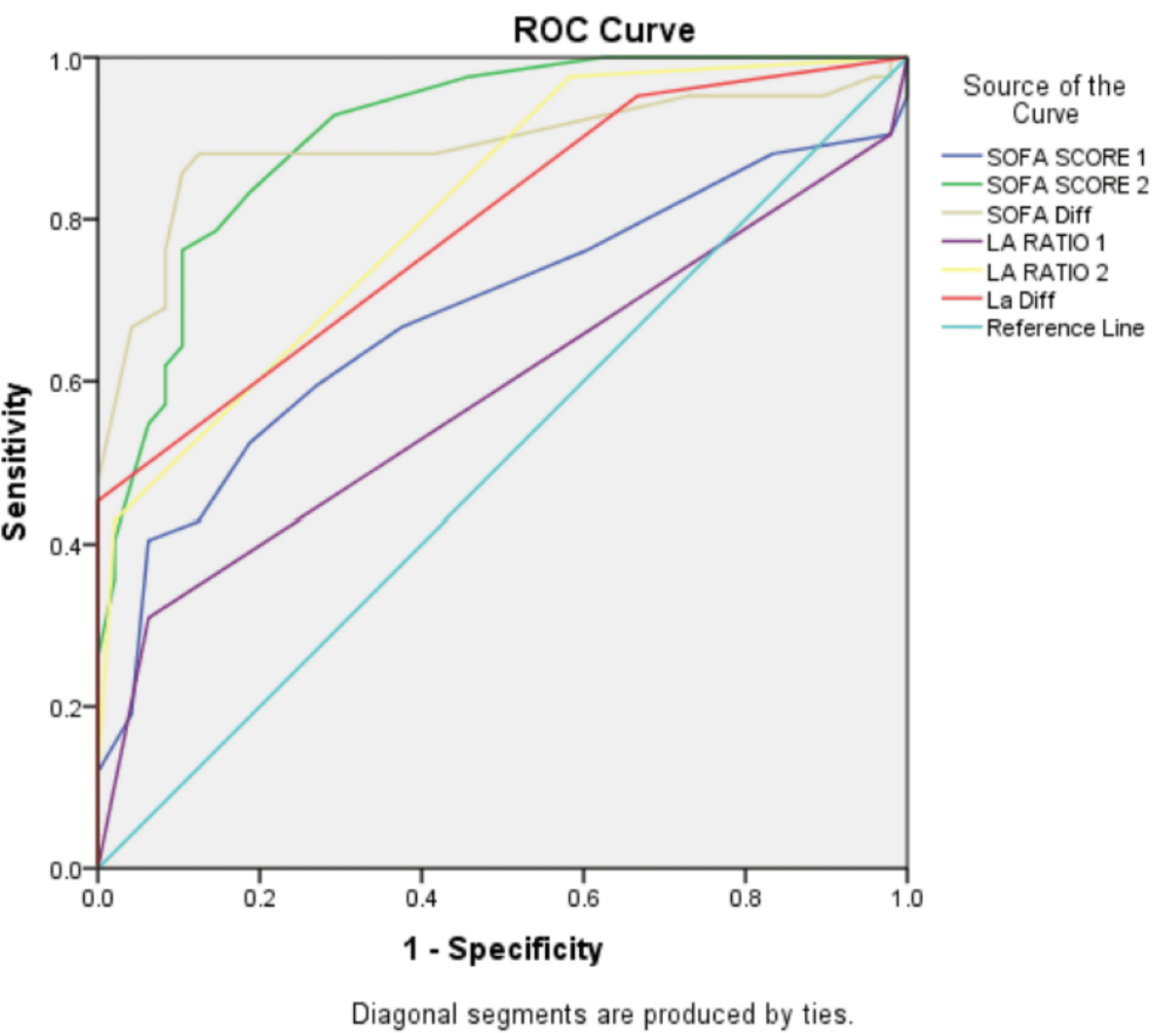
ROC curve analysis demonstrating the predictive performance of the SOFA score and L/A ratio parameters for in-hospital mortality among patients with sepsis The analysis includes the SOFA score on days one and three; the SOFA difference, i.e., day three score – day one score; the L/A ratio on days one and three; and the L/A difference on day three – day one. The diagonal reference line represents the performance of a non-discriminatory test (AUC = 0.5). Higher AUC values indicate better discriminatory ability for predicting mortality. ROC: Receiver operating characteristic; SOFA: Sequential organ failure assessment; L/A: Lactate/albumin; AUC: Area under the curve

The ROC analysis showed that the SOFA Score on day three had excellent predictive ability for mortality (AUC = 0.907). The SOFA difference also demonstrated strong discrimination (AUC = 0.891). The L/A ratio on day three showed good predictive performance (AUC = 0.813), while the L/A difference had moderate discrimination (AUC = 0.794). Baseline L/A ratio had poor predictive value (AUC = 0.586) (Table 6 and Figure 1).

**Table 6:** ROC curve analysis for mortality prediction Discriminatory ability of each variable is expressed as AUC. ROC: Receiver operating characteristic; AUC: Area under the curve; SOFA: Sequential organ failure assessment; L/A: Lactate/albumin

Variable	AUC
SOFA score 1	0.682
SOFA score 2	0.907
SOFA difference	0.891
L/A ratio 1	0.586
L/A ratio 2	0.813
L/A difference	0.794

On day one, the majority of patients were not ventilated (65, 72.2%); 16 (17.8%) were intubated, and nine (10%) received non-invasive ventilation (NIV). Intubated patients had the highest mean SOFA score (8.94 ± 3.53) compared to NIV (5.78 ± 2.82) and non-ventilated patients (7.35 ± 3.03), with a statistically significant difference (H = 7.210, p = 0.027). However, the L/A ratio did not differ significantly across groups on day one (p = 0.326). By day three, intubated patients increased to 25 (27.8%), while 57 (63.3%) required no ventilation. The mean SOFA score was highest in intubated patients (11.96 ± 4.61) and lowest in those without ventilation (4.86 ± 4.01), showing a highly significant difference (H = 30.848, p < 0.001). Similarly, the L/A ratio was significantly elevated among intubated patients (1.44 ± 0.74) compared to NIV (1.22 ± 1.02) and non-ventilated patients (0.88 ± 0.83) on day three (H = 14.523, p = 0.0001). Table 7 shows the comparison of the SOFA score and L/A ratio per the ventilation status on days one and three.

**Table 7:** SOFA score and L/A ratio per ventilation status on day one and day three Categorical data are presented as numbers (n) and percentages (%), and continuous variables as mean ± SD. The Kruskal–Wallis test was applied. * A p-value < 0.05 was considered statistically significant. SOFA: Sequential organ failure assessment; L/A: Lactate/albumin; NIV: Non-invasive ventilation

Variable	Ventilation status	Day one		Day three	
		N (%)	Mean ± SD	N (%)	Mean ± SD
SOFA Score	Intubated	16 (17.8)	8.94 ± 3.53	25 (27.8%)	11.96 ± 4.61
	NIV	9 (10%)	5.78 ± 2.82	8 (8.9%)	8.50 ± 3.93
	Nil	65 (72.2%)	7.35 ± 3.03	57 (63.3)	4.86 ± 4.01
Kruskal-Wallis H value		7.210		30.848	
p-value		0.027*		< 0.001*	
L/A ratio	Intubated	16 (17.8)	0.97 ± 0.53	25 (27.8%)	1.44 ± 0.74
	NIV	9 (10%)	0.99 ± 0.40	8 (8.9%)	1.22 ± 1.02
	Nil	65 (72.2%)	1.08 ± 0.43	57 (63.3)	0.88 ± 0.83
Kruskal-Wallis H value		2.239		14.523	
p-value		0.326		0.0001*	

On day one, 48 (53.3%) patients required no vasopressor support, 36 (40%) received a single inotrope, and six (6.7%) required multiple inotropes. Although the mean SOFA score increased progressively from nil (6.77 ± 2.88) to multiple inotropes (9.83 ± 5.27), the difference was not statistically significant (p = 0.099). The L/A ratio also showed no significant difference across groups on day one (p = 0.574). By day three, 50 (55.6%) patients required no vasopressors, 24 (26.7%) received a single inotrope, and 16 (17.8%) required multiple inotropes. Patients on multiple inotropes demonstrated the highest SOFA scores (12.81 ± 2.64) and L/A ratios (2.11 ± 1.26), whereas those without vasopressor support had the lowest values (SOFA 3.72 ± 2.67; L/A 0.65 ± 0.29). Both SOFA score (H = 47.583, p < 0.001) and L/A ratio (H = 36.989, p < 0.001) showed highly significant differences across vasopressor groups on day three (Table 8).

**Table 8:** SOFA score and L/A ratio per vasopressor status on day one and day three The Kruskal–Wallis test was applied. *A p-value < 0.05 was considered statistically significant. SOFA: Sequential organ failure assessment; L/A: Lactate/albumin

Variable	Ventilation status	Day one		Day three	
		N (%)	Mean ± SD	N (%)	Mean ± SD
SOFA score	Nil	48 (53.3)	6.77 ± 2.88	50 (55.6)	3.72 ± 2.67
	Single inotrope	36 (40)	8.03 ± 2.94	24 (26.7)	10.54 ± 5.00
	Multiple inotropes	6 (6.7)	9.83 ± 5.27	16 (17.8)	12.81 ± 2.64
Kruskal-Wallis H value		4.619		47.583	
p-value		0.099		< 0.001*	
L/A ratio	Nil	48 (53.3)	1.00 ± 0.42	50 (55.6)	0.65 ± 0.29
	Single inotrope	36 (40)	1.08 ± 0.44	24 (26.7)	1.24 ± 0.66
	Multiple inotropes	6 (6.7)	1.26 ± 0.71	16 (17.8)	2.11 ± 1.26
Kruskal-Wallis H value		1.110		36.989	
p-value		0.574		< 0.001*	

## Discussion

Sepsis has a reported mortality ranging from 20% to 40%, depending on disease severity and healthcare infrastructure [14]. In the present study, the in-hospital mortality rate was 42 patients (46.7%), which is comparable to Indian ICU findings such as Poriya et al. (41.4%) [15]. The predominance of elderly patients (41, 45.6%) aged 61 to 80 years and males (55, 61.1%) is consistent with observations reported by Krishnamurthy et al. and Nazeer et al. [16,17]. These findings suggest that our cohort reflects the demographic pattern commonly seen in sepsis populations in similar healthcare settings.

Although the baseline L/A ratio did not significantly differ between survivors and non-survivors (p = 0.625), the day three L/A ratio and L/A difference were significantly higher in non-survivors (p < 0.001). This highlights the importance of serial assessment rather than reliance on a single time point value. Poriya et al. demonstrated that the L/A ratio outperformed the SOFA score in predicting mortality (AUC 0.932 vs. 0.857) [15]. Similarly, Bajaj et al. reported superior discrimination of the L/A ratio (AUC 0.943) compared to SOFA (AUC 0.816) [18]. A pooled meta-analysis by Yoon et al. showed moderate predictive value of the L/A ratio (pooled AUC 0.74), while Chebl et al. reported that the L/A ratio was superior to lactate alone in predicting mortality [19,20]. In contrast, Chen et al. observed modest performance (AUC 0.61), suggesting that predictive strength may vary depending on the timing of measurement, disease severity, and patient characteristics [21]. Overall, our findings align more closely with recent Indian ICU studies where serial L/A measurement demonstrated strong prognostic ability [15,18].

With respect to the SOFA score, our results reaffirm its established prognostic utility. Day one SOFA score was significantly higher in non-survivors, indicating better early discrimination compared to baseline L/A ratio. By day three, both the SOFA score and L/A ratio showed excellent separation between survivors and non-survivors (p < 0.001). The ROC analysis in our study demonstrated strong predictive ability for SOFA day three (AUC 0.907) and SOFA difference (AUC 0.891), which was comparable to L/A day three (AUC 0.813). Karim et al. also reported a significant correlation between L/A ratio and SOFA score at admission (r = 0.726, p < 0.001) [22]. These findings suggest that although SOFA remains a comprehensive and validated tool, the L/A ratio offers a simpler and rapidly obtainable alternative with comparable prognostic value, particularly when assessed dynamically.

Another important observation was the association of the day three L/A ratio with organ support requirements. Patients requiring mechanical ventilation (33, 36.7%) and multiple vasopressors (16, 17.8%) had significantly higher day three L/A ratios. Nazeer et al. reported an AUC of 0.904 for the L/A ratio in predicting mechanical ventilation [17], while Poriya et al. demonstrated a strong correlation between the L/A ratio and the cardiovascular component of the SOFA score [15]. Wang et al. showed that the elevated L/A ratio predicted shock and respiratory failure, and Makram et al. reported a strong association between a higher L/A ratio and refractory shock in pediatric ICU patients [23,24]. These consistent findings across different settings indicate that the L/A ratio reflects both tissue hypoperfusion (via lactate) and systemic inflammatory burden with capillary leak (via albumin), making it biologically plausible as a marker of circulatory and respiratory failure severity.

Regarding hospital stay, most patients (77, 85.6%) had a duration of <14 days. Unlike the study by Poriya et al., which reported a positive correlation between a higher L/A ratio and prolonged ICU stay [15], our study did not observe a strong association with baseline values. This difference may be attributed to the dichotomous categorization of hospital stay rather than continuous analysis as well as the focus on early measurements. Overall, our findings strengthen the growing body of evidence that serial L/A ratio measurement provides clinically meaningful prognostic information comparable to the SOFA score, particularly for mortality prediction and organ support requirements, with special relevance in resource-limited ICU settings.

Strengths and limitations

The present study has several strengths. It provides a direct comparison between the L/A ratio and the SOFA score in predicting mortality and organ support requirements for sepsis. Serial measurements allowed assessment of biomarker trajectory, which improved prognostic interpretation beyond single time-point analysis. The inclusion of clinically relevant outcomes such as mortality, mechanical ventilation, vasopressor requirement, and hospital stay enhances the practical applicability of the findings, particularly in resource-limited ICU settings. However, there are certain limitations; this is a single-center study with a small sample size, short follow-up duration, and restricted long-term outcome assessment. Additionally, specific cutoff values for the L/A ratio were not derived, and lactate clearance was not evaluated, which could have provided further prognostic insights.

## Conclusions

The L/A ratio is a simple and useful marker to assess prognosis in patients with sepsis. It combines two important laboratory values, serum lactate, which reflects tissue hypoperfusion, and serum albumin, which reflects systemic inflammation and overall physiological status, into one measure of disease severity. Serial assessment of the L/A ratio provides greater prognostic value than a single measurement, as rising or falling trends can indicate whether a patient is improving or deteriorating. When used along with established scoring systems such as the SOFA score, serial L/A measurements may enhance early risk stratification and help identify patients at higher risk of adverse outcomes. Because it is easy to calculate from routine laboratory tests, the L/A ratio can be readily incorporated into routine sepsis evaluation to support timely clinical decision-making, particularly in resource-limited settings.
